# CD33 forms functional dimers on cell surface to modulate Alzheimer risk

**DOI:** 10.1002/alz70855_101544

**Published:** 2025-12-23

**Authors:** Ye Zhou, Kanayo Satoh, Roger B. Dodd, Masahiro Enomoto, Yalun Zhang, Fusheng Chen, Beatrice Acheson, Deniz Ghaffari, Jennifer K Griffin, Wesley Asher, Jamie Manning, George Dukas, Jonathan Javitch, Mamunur Rashid, Seema Qamar, Jean Sevalle, Christopher Bohm, Paul E Fraser, Elizabeth M Bradshaw, Peter St George‐Hyslop

**Affiliations:** ^1^ University of Toronto, Toronto, ON, Canada; ^2^ Cambridge University, Cambridge, Cambridge, United Kingdom; ^3^ University Health Network, Toronto, ON, Canada; ^4^ Columbia University, New York, NY, USA; ^5^ Department of Neurology, Columbia University, New York, NY, USA

## Abstract

**Background:**

The sialic‐acid binding immunoglobulin‐like lectin 3 receptor (Siglec‐3 */ CD33)* is one of the highly associated AD risk genes. Previous studies revealed that the non‐coding AD‐risk alleles (rs3865444 and rs12459419) are associated with increased total levels of CD33 expression and a higher relative expression of the long CD33^M^ splice form. However, the molecular basis of the immuno‐inhibitory function of CD33 remains unclear.

**Method:**

To confirm the presence of CD33 dimers, we conducted multiple experiments. Blue Native Gel electrophoresis and co‐immunoprecipitation assays were used to detect CD33 bands corresponding to the expected molecular weights. Flow cytometry with specific antibodies was performed to quantify cell‐surface CD33. Additionally, single‐molecule fluorescence resonance energy transfer (smFRET) combined with TIRF imaging was employed to visualize CD33 dimers on the cell surface. Furthermore, Western Blotting of phosphorylation of CD33 and its downstream molecule were performed to verify if the dimers were functional.

**Result:**

Biochemical analyses demonstrated that CD33^M^ and CD33^m^ can form homodimers or heterodimers. Flow cytometry confirmed that CD33^M^ isoforms are selectively trafficked to the cell surface, while smFRET imaging verified the presence of dimers on the cell surface. The elevation of the CD33 pathway following stimulation with CD33‐specific ligands provided evidence that CD33^M^ homodimers are functional.

**Conclusion:**

This study reveals the critical role of CD33^M^ in AD pathology by elucidating its molecular mechanisms. We provide direct evidence that CD33^M^ and CD33^m^ isoforms can form both homodimers and heterodimers. However, only CD33^M^ isoforms are preferentially trafficked to the cell surface and form functional dimers. These findings advance our understanding of the molecular basis of CD33's immuno‐inhibitory function and offer new insights into its involvement in AD risk, potentially paving the way for the development of targeted therapeutic strategies.

